# *Moringa oleifera* Leaves as Eco-Friendly Feed Additive in Diets of Hy-Line Brown Hens during the Late Laying Period

**DOI:** 10.3390/ani11041116

**Published:** 2021-04-13

**Authors:** Ahmed A. A. Abdel-Wareth, Jayant Lohakare

**Affiliations:** 1 Department of Animal and Poultry Production, Faculty of Agriculture, South Valley University, Qena 83523, Egypt; a_bkr1@yahoo.com or; 2 Department of Agriculture-Animal Science option, University of Arkansas at Pine Bluff, Pine Bluff, AR 71601, USA

**Keywords:** blood biochemicals, egg production, egg quality, laying hens, *Moringa oleifera*

## Abstract

**Simple Summary:**

It is of the utmost importance to explore the merit of a new phytogenic feed additive for sustainable egg production in laying chickens during the late laying period. Thus, the current study was designed to evaluate the effect of the addition of *Moringa oleifera* leaves to laying hen diets on laying performance, egg quality, excreta ammonia concentrations and blood biochemical parameters. The findings showed that the egg production, weight and mass and eggshell quality of laying hens fed with *Moringa oleifera* leaves during the late laying period were significantly improved in comparison with those of the hens in the control group. *Moringa oleifera* leaves supplementation decreased excreta ammonia concentration and serum cholesterol and triglycerides as well as serum liver enzymes, uric acid, and creatinine levels compared to those of the control group.

**Abstract:**

This study investigated the dietary effects of *Moringa oleifera* leaves supplementation on egg quality, laying performance, excreta ammonia concentrations and serum biochemistry of laying chickens during the late laying period. A total of 240 64-week-old Hy-Line Brown hens were assigned to four treatment diets including *Moringa oleifera* leaves at 0, 3, 6 or 9 g/kg, respectively, for eight weeks. The treatments had twelve replicates with five hens each. The results revealed that incremental dietary *Moringa oleifera* leaves significantly increased (*p* < 0.01) egg weight, production, and mass through 64–68, 68–72 and 64–72 weeks of age. Simultaneously, feed conversion ratio was significantly improved (*p* < 0.01) with *Moringa oleifera* leaves supplementation compared with the control. Haugh units and the thickness of eggshells significantly improved as a response to diets supplemented with 3, 6 and 9 g/kg *Moringa oleifera* leaves at 72 weeks of age. Interestingly, excreta ammonia concentrations, serum cholesterol, aspartate transaminase and alanine aminotransferase significantly decreased by *Moringa oleifera* leaves supplementation compared with the control group. In conclusion, introducing *Moringa oleifera* leaves supplementation at 3, 6 and 9 g/kg increased egg production, eggshell quality, Haugh units, and decreased serum cholesterol, triglycerides, excreta ammonia concentrations besides serum liver enzymes, uric acid and creatinine. Overall, based on the observed results, *Moringa oleifera* leaves supplementation was very promising and these leaves could be used as an effective feed additive in laying hens’ diet during the late laying period.

## 1. Introduction

Natural herbal plants received much more attention as alternative to antibiotics growth promoters in the nutrition of laying hens to improve the egg production. The use of in-feed antibiotics may affect birds, human health, and the environment due to their cross and multiple resistances to bacterial infections in humans. However, it is still a common practice in the Middle East, particularly in Egypt, to add antibiotics as growth promoters to poultry diets to improve their production, health, and egg and meat quality. Phytogenics are one of the explored alternatives to feed antibiotics in laying hens because of their antimicrobial and antioxidant activities and positive effects on productive performance [1,2,3]. Some studies illustrated and proved phytogenic applications in poultry nutrition [4,5,6,7,8,9,10] to be beneficial in enhancing productive performances and health status of laying hens. Higher excreta ammonia production reduces the productive and health status of chickens [11,12], increases chicken mortalities by increasing susceptibility of birds to infections [11,13], impair human health and act as a major aerial pollutant [14,15,16]. An adequate nutritional strategy is necessary to diminish ammonia concentrations and to increase egg production and egg quality in laying hens. Previous studies have demonstrated the suitability of different phytogenics in the nutrition of laying hens [6,7,10], however, information about *Moringa* as a feed additive in laying hens are insufficient and require further studies.

*Moringa oleifera* has many common names such as moringa, horseradish tree, drumstick tree, miracle tree, ben oil tree or benzoil tree. *Moringa oleifera* has a broad use in herbal medicine, believed to be beneficial in immunity enhancement and having antimicrobial and antioxidant traits and a hypo-cholesteremic effect on chickens, mainly due to its nutritional and therapeutic values [17,18,19]. Briones et al. [20] found that the dietary *Moringa* leaves supplementation in layers diet can improve production performance and egg quality. Likewise, dietary supplementation of *Moringa oleifera* at 5% improved yolk color, albumen height and Haugh unit of eggs [21]. The results of the previous studies in layers using *Moringa oleifera* could be attributed to dose, feeding duration, composition, processing method, environmental stress aspects or birds’ age. Furthermore, it remains vague whether specific effects are resulting from herbal leaves or a synergistic effect due to multiple components [17]. More studies are needed in detecting the optimum levels of *Moringa oleifera* as feed additive to sustain egg production of laying hens at the late laying period. Therefore, this study was designed to assess the impact of dietary supplementation of different levels of *Moringa oleifera* leaves on laying performance, egg quality, excreta ammonia concentration and blood biochemical parameters of laying hens in the late laying period.

## 2. Materials and Methods 

### 2.1. Experimental Birds, Design and Feed Preparation 

A total of 240 (average weight = 1860 ± 100 g, 64-week-old) Hy-Line Brown hens were assigned to four treatment diets including *Moringa oleifera* leaves at 0, 3, 6 and 9 g/kg, respectively, for eight weeks. The required amount of *Moringa oleifera* leaves were added to a small amount of basal feed and mixed. The mixture was then added to the preset amount of feed for each treatment and mixed thoroughly before being fed to each group. Each treatment had 12 replicates with five hens each. 

The hens were kept in 48 cages with 60 × 60 × 40 cm^3^ dimensions. Each cage was provided with feeders and water nipples with a light program of 16 hours of light every day, and the temperature was maintained at approximately 24 °C. Water and feed were provided for ad libitum consumption. The basal diets were calculated according to the breeder’s manual for Hy-Line Brown and the recommendations of National Research Council (NRC) [22]. The birds were fed this mash form of diets for 64–72 weeks of age. The basal experimental diet was formulated as in Table 1.

### 2.2. Diet Analysis and Preparation of Dried Moringa oleifera Leaves 

Feed samples were analyzed including moisture (Method Nr. 930.15), ash (Method Nr. 942.05), protein (Method Nr. 984.13), ether extract (Method Nr. 954.02), calcium (Method Nr. 927.02) and phosphorous (Method Nr. 935.59) as described by the AOAC International [23]. The gross energy was determined using adiabatic bomb calorimetry (Parr Instrument Company, Moline, IL, USA). The fresh excreta of each replicate were used for ammonia analysis. Ammonia-N (NH_3_-N) concentration was estimated by the distillation method according to AOAC [23] on fresh excreta samples. Dried *Moringa oleifera* leaves were purchased from Qena, Egypt, from a local herbalist. The *Moringa oleifera* essential oils were extracted by hydro-distillation in a Clevenger-type apparatus for three hours and were used for analysis. The analysis of *Moringa oleifera* oils took place via a gas chromatography-mass spectrometry instrument [6]. The gas chromatography-mass spectrometry (GC-MS) analysis of the extract was carried out using instrument having a TRACE GC Ultra Gas Chromatographs (THERMO Scientific Corp., USA), coupled with a THERMO mass spectrometer detector (ISQ Single Quadrupole Mass Spectrometer). The GC-MS system was equipped with a TG-5MS column (30 m × 0.25 mm i.d., 0.25 μm film thickness). Helium was utilized in the analyses as carrier gas at a flow rate of 1.0 mL/min and a split ratio of 1:10 with the following temperature program: 60 °C for 1 min, rising at 3.0 °C/min to 240 °C and held for 1 min. The injector and detector were held at 240 °C. Diluted samples (1:10 hexane, v/v) of 0.2 µL of the mixtures were injected. Mass spectra were obtained by electron ionization (EI) at 70 eV, with a spectral range of m/z 40–450. Most of the compounds were identified with the analytical method: mass spectra (authentic chemicals, Wiley spectral library collection and NSIT library). The active compounds in the Moringa leaves are presented in Table 2. 

### 2.3. Production Performance Parameters

During the experimental period, the eggs number and weight from each replicate were measured every day. Egg production was calculated as average hen-day egg production. The consumption of the feed was measured weekly. The Feed conversion ratio (FCR) was calculated as the weight of feed intake divided by egg mass per replicate cage. Egg mass was calculated as: egg weight in grams × egg production rate. The variables were measured per replicate cage. 

### 2.4. Egg Quality

The egg quality parameters were assessed at 68 and 72 weeks of age. A total of 36 eggs during each period per treatment (3 eggs per replicate) were used to assess the egg quality on the same day [15]. The collected eggs were weighed and broken onto a glass break-out table for albumen and yolk measurements. The dimensions of yolks and albumen were measured using calipers and weighed. Eggshell weights, albumen and yolk were measured and expressed as percentages of the whole egg’s weight. Eggshell thickness (without inner and outer shell membranes) was gauged at the middle of the eggshell using a QCT shell thickness micrometer (Technical Services and Supplies Ltd., England). Haugh Units (HU) was calculated from the records of egg weight (W) and albumen height (H) through the following formula: HU = 100 log10 (H − 1.7W^0.37^ + 7.56), according to Haugh [24].

### 2.5. Blood Sampling and Laboratory Analyses

After the experimental period, blood samples were collected from the wing vein of 3 birds per replicate (a total of 36 blood samples, per treatment) in tubes to determine the serum biochemical indices such as total cholesterol, triglycerides, liver enzymes such as aspartate transaminase (AST), Alanine aminotransferase (ALT), uric acid and creatinine spectrophotometrically via the matching kits as per the instructions of the manufacturer (Spectrum chemical company, Obour City- Cairo, Egypt). 

### 2.6. Statistical Analysis

All analyses were performed using the general linear model (GLM) procedures of SAS 9.2 [25]. The model only included the level of *Moringa oleifera* leaves supplementation. The cages were the experimental units for all analyses. The impacts of *Moringa oleifera* levels were analyzed via orthogonal polynomial contrasts for linear and quadratic effects. Differences were deemed to be statistically significant at *p* < 0.05, and data are expressed as mean and pooled SEM.

## 3. Results

### 3.1. Productive Performance and Ammonia Concentrations

Throughout the experimental period no mortalities were recorded, and the birds were healthy. The incremental dietary *Moringa oleifera* leaves at 3, 6 and 9 g/kg significantly enhanced (*p* < 0.01) egg production, weight and mass compared with the control group during 64–68, 68–72 and 64–72 weeks of age (Table 3). Furthermore, the feed conversion ratio was linearly and quadratically decreased (*p* < 0.01) with the increasing levels of *Moringa oleifera* leaves at 3, 6 and 9 g/kg during 64–68, 68–72 and 64–72 weeks (Table 3). Interestingly, excreta ammonia concentrations significantly decreased (*p* < 0.001) due to *Moringa oleifera* supplementations (Table 3). Within the entire interval of 64–72 weeks, supplementation of *Moringa oleifera* leaves to laying hens diets at 3, 6 and 9 g/kg significantly improved (*p* < 0.001) feed conversion ratio by 8.26, 12.11 and 8.83% in comparison with control, respectively.

### 3.2. Egg Quality

Table 4 shows the effects of dietary *Moringa oleifera* leaves supplementation on egg quality. Hens fed *Moringa oleifera* leaves at levels of 3, 6 and 9 g/kg had increased eggshell thickness, eggshell percentage and Haugh units than those fed the control treatment at 68 and 72 weeks of age (*p* < 0.01). However, *Moringa oleifera* leaves supplementation did not influence other egg quality characteristics like albumen and yolk percentages. 

### 3.3. Serum Biochemical Parameters

Table 5 shows the effects of *Moringa oleifera* leaves supplementation on serum biochemicals. According to the serum metabolic profile, serum cholesterol and triglycerides significantly decreased (*p* < 0.001) with the increase of *Moringa oleifera* leaves levels in laying hens’ diets. Additionally, serum enzymes (AST and ALT) were less (*p* < 0.001) in hens fed *Moringa oleifera*—supplemented diets. Moreover, serum creatinine and uric acid decreased (*p* < 0.001) in hens fed *Moringa oleifera*—supplemented diets.

## 4. Discussion

Few studies are available in the literature on the application of *Moringa oleifera* as a potential alternative feed additive for laying hens during the late laying period; therefore, the comparison was made with other studies that used essential oils or herbs. The main determinants of the active components of *Moringa oleifera* leaves—prior to the addition to the hen diets—could prove useful for insights on its effect on productive performances. In this study, *Moringa oleifera* leaves’ active compounds were consistent with those observed in previous studies [19,26]. Furthermore, β-sitosterol and 4-[α-(L-rhamnosyloxy) benzyl]-o-methyl thiocarbamate (trans) are two important active compounds presence in the leaves essential oil of *Moringa oleifera* that exhibit health-promoting properties [26,27]. Mahfuz and Pioa [27] contributed a review on Moringa oleifera as a natural feed supplement in laying hens’ diet. It was concluded that the application of Moringa oleifera in poultry nutrition enhanced the productive performance and health, however, the inclusion levels of Moringa oleifera in poultry diet and their mode of actions are still unclear and need further consideration. 

Overall, in the present study, egg production, egg mass and feed conversion ratio improved with the addition of *Moringa oleifera* leaves to diets, compared with the control group of laying hens. The higher egg weight, mass and production are potentially attributed to the specific *Moringa oleifera* leaves’ active components (Table 2). Moreover, the improvements in the egg production could be due to *Moringa oleifera* leaves’ reported antimicrobial and antioxidant properties, and gastro-protective and mucus-enhancing enzyme activities; this is resulting from its essential oil and active components [18,19,27]. Similarly, supplementation of *Moringa oleifera* leaves meal at 10 g/kg improved feed conversion ratio and hen-day egg production of laying hens at 55 weeks of age [28], in accordance with the dose and results in our study. On the other hand, the addition of *Moringa oleifera* at 10% significantly increased egg production of laying hens [29], a ten-times higher dose than the present study. The hen-day egg production, weight and mass and feed conversion ratio were significantly improved by *Moringa oleifera* fresh leaves as feed supplement compared with the control group [30]. On the contrary, this study contradicts Ashour et al.’s [31], who reported that dietary supplementations of *Moringa oleifera* seeds, leaves and their combination did not significantly influence (*p* < 0.05) feed intake, conversion ratio and egg weight compared with the control. Moreover, Olugbemi et al. [32] reported that feed intake, feed conversion ratio egg production was not influenced by the inclusion of 5% and 10% Moringa oleifera leaves in laying hen diets. The maximum percentage that was used in our study was 0.9% far lower than the other reported studies. The inconsistencies in the results of these studies may be due to the use of different bird strains, age, animal production stage, plant parts, agroclimatic conditions, herb compositions, addition levels, application and cultivation methods [3,5,17]. There are also studies that used variable doses of supplementation and used plant parts, such as seeds, leaves, extracts, or active compounds. In short, many researchers reported that supplementation levels of *Moringa oleifera* ranging from 0.5% up to 10% could have a positive response in enhancing the productive performance and health status of laying hens [28,29,30]. Further studies are still needed to determine the optimal level of supplementation that increase productive performance in chickens, considering the economic benefits and health status.

Ammonia is derived from the degradation of nitrogenous feed ingredients and is a colorless gas with pungent odor. Ammonia is considered as one of the harmful gases as it has negative effects on poultry including increased susceptibility to respiratory diseases and therefore reduced productive performance, besides poses a threat to the workers’ health. In the current study, excreta ammonia concentrations significantly decreased with the increasing levels of *Moringa oleifera* leaves supplementation. The phytogenic functions might have stimulated the intestinal enzyme activity, reduced pathogenic bacteria, increased nitrogen absorption and controlled secretion of odor and ammonia content [33]. Moreover, Hong et al. [34] reported that ileal ammonia concentration was significantly decreased in broiler chickens fed 125 mg/kg essential oil (essential oil derived from oregano, anise, and citrus peel). Supplementation of 100 mg/kg of yucca in the laying-hen diet significantly reduced ammonia generation and emission in laying-hen barns [35]. 

Egg quality is considered as one of the most important criteria in the egg industry, influencing economic profitability. Hens fed *Moringa oleifera* leaves at 3, 6 and 9 g/kg had increased eggshell thickness, percentage and Haugh units compared to those ingesting the control treatment at 68 and 72 weeks of age (Table 4). It may be hypothesized that *Moringa oleifera* allows increased eggshell weight and thickness due to the phytogenic compounds that may have the ability to improve calcium storage, uterine functions and intestinal secretions, which could lead to improving eggshell and egg quality [10,36]. Furthermore, the eggshell and Haugh units are vital for the egg industry and are considered as signs of egg freshness and optimal shelf life [6,37,38]. The active components of *Moringa oleifera* leaves seem to improve oviposition and uterine health, thus improving egg quality. In accordance with our results, Briones et al. [20] stated that *Moringa oleifera* leaves can be supplemented to layers’ diets to improve egg quality. Ahmad et al. [39] also reported that supplementation of *Moringa oleifera* leaves at 5, 10 and 15 g/kg significantly increased Haugh units and eggshell thickness of laying hens, from 50–66 weeks of age. In contrast, Abou-Elezz et al. [30] surmised that *Moringa oleifera* leaves in diets did not affect egg quality parameters, including shell percent and thickness. 

Since the studies on this subject are few, the full comprehension and discussion of physiological responses of laying hens to *Moringa oleifera* leaves are hindered. In the current study, *Moringa oleifera* leaves have shown important impacts on serum cholesterol, triglycerides, AST, ALT, creatinine and uric acid of laying hens during the late laying period; however, the exact mechanism of *Moringa* leaves on blood metabolic profiles are not known yet [40]. The serum values of the measured biochemicals are within the normal physiological range [21,38,41,42]. Furthermore, Ghasi et al. [43] reported that *Moringa oleifera* leaves have hypo-cholesterolemic functions. *Moringa oleifera* leaves exhibit cholesterol-lowering activities affected by the active compounds present in them and these compounds could decrease the intestine dietary cholesterol uptake [44,45]. Supplementation of *Moringa oleifera* leaves to broiler diets significantly lowered serum cholesterol contents [46,47]. Ahmad et al. [39] reported that supplementation of *Moringa oleifera* leaves at 5, 10 and 15 g/kg significantly lowered serum cholesterol and uric acid of laying hens, from 50–66 weeks of age. In the current study, hens fed a diet supplemented with *Moringa oleifera* leaves had significantly reduced serum AST, ALT, uric acid and creatinine compared with the control group (Table 5), considered as a positive sign of the liver and kidney’s synthetic functions and laying hens’ health during the late period. For liver functions, ALT and AST are the most widely used clinical biomarkers for hepatic health and leakage of ALT and AST from the hepatocytes into the blood occurs following hepatocellular injury and their levels usually rise whenever the liver is being damaged [47]. Therefore, it could be reported that supplementation of *Moringa oleifera* leaves promote liver health by decreasing serum ALT and AST concentrations in laying hens. These results concur with Halaby et al. [48] who reported that *Moringa oleifera* leaves significantly decreased serum uric acid and creatinine in rats more than that of the positive control group. Supplementation of *Moringa oleifera* leaves at 0.5, 1.0, 1.5 and 2.0% induced a mild reduction in cholesterol and uric acid, AST and ALT in broiler chickens [49], implicating that there was no liver damage. Contrarily, laying hens fed the diets containing 15% *Moringa oleifera* leaves showed surged AST activity more than those fed the control diet [21]. Multiple reports disagree on the influence of supplemental *Moringa oleifera* on liver and kidney functions, which could be due to the concentration of the active substances in their *Moringa* sources. Further studies focusing on the actual mechanisms of the active compounds are warranted.

## 5. Conclusions

In conclusion, *Moringa oleifera* leaves supplementation at 3, 6 and 9 g/kg increased egg production, eggshell quality, Haugh units, and decreased serum cholesterol, triglycerides, excreta ammonia concentrations besides serum liver enzymes, uric acid and creatinine, and could be used as an effective feed additive in laying hens’ diet during the late laying period. Further studies are still needed to elucidate the exact mechanism of action of Moringa leaves and their essential oils and to specify the effective dose in poultry nutrition. 

## Figures and Tables

**Table 1:** Ingredient composition and chemical analysis of the basal diet (in percent).

Ingredients	%
Yellow corn	68.15
Soybean meal 44% CP	17.79
Soybean oil	0.81
Corn gluten 60% CP	2.5
Di-calcium phosphate	1.92
Limestone	7.97
Premix *	0.21
Dl-Methionine	0.25
Choline chloride (50%)	0.4
Total	100
Determined analysis,	%
Dry matter	89
Ash	11.8
Crude protein	18.5
Ether extract	4.21
Calcium	3.73
Phosphorus	0.58
Gross energy (MJ/kg)	16.3

* The following was appropriated per kg of diet: vitamin A, 12,000 IU; vitamin D_3_, 7200 IU; vitamin E, 20 IU; vitamin K, 3 mg; vitamin B_1_, 833 IU; vitamin B_2_, 2000 IU; vitamin B_12_, 60 IU; pyridoxine, 0.225 µg; pantothenic acid, 10 mg; niacin, 35 mg; folic acid, 1.5 mg; biotin 125 mg; Mn, 90 mg; Cu, 7.5 mg; Zn, 65 mg; Fe, 50 mg; Se, 0.1 mg.

**Table 2:** The main compounds in the *Moringa* leaves.

Retention Time	g/100 g Essential Oil	Compounds
17.75	0.71	thymol
25.14	1.68	α-curcumene
26.17	0.40	β-bisabolene
26.82	1.6	β-sesquiphellandrene
29.05	0.45	caryophyllene oxide
29.16	1.03	p-cymene
30.14	0.94	trans-nuciferol
30.42	5.51	hexacosane
31.11	0.63	7-epi-cis-sesquisabinene hydrate
32.55	55.46	Ar-turmerone
32.63	5.73	tumerone
33.84	13.94	curlone
35.33	0.89	heptacosane
38.54	1.88	6-dodecanone
40.93	0.97	farnesyl acetone
47.11	1.33	phytol
55.76	3.23	14-β-H-PREGNA
57.28	3.62	tetrapentacontane, 1,54-dibromo

**Table 3:** The effect of *Moringa oleifera* leaves on the productive performance of laying hens during the late laying period.

Items	*Moringa oleifera* Leaves (g/kg)				SEM ^1^	*p* Value	
	0	3	6	9		Linear	Quadratic
64–68 weeks of age							
Egg weight, (g/hen/day)	60.89	63.58	64.75	63.49	0.57	0.008	0.009
Egg mass, (g/hen/day)	49.46	53.47	56.02	54.41	0.61	<0.001	0.002
Hen-day production, (%)	81.24	84.09	86.53	85.67	0.81	0.003	0.042
Feed intake, (g/hen/day)	105.6	103.7	106.0	105.3	1.01	0.773	0.532
Feed conversion ratio	2.136	1.939	1.893	1.937	0.03	0.002	0.006
68–72 weeks of age							
Egg weight, (g/hen/day)	60.89	63.23	65.92	63.98	0.42	<0.001	0.001
Egg mass, (g/hen/day)	49.64	53.37	57.46	54.81	0.29	<0.001	0.001
Hen-day production, (%)	81.52	84.42	87.18	85.67	0.53	<0.001	0.003
Feed intake, (g/hen/day)	105.7	102.6	105.3	105.0	1.08	0.894	0.252
Feed conversion ratio	2.128	1.923	1.833	1.916	0.02	0.001	0.001
64–72 weeks of age							
Egg weight, (g/hen/day)	61.43	63.93	65.37	64.31	0.45	0.001	0.005
Egg mass, (g/hen/day)	50.70	54.15	57.67	55.29	0.62	<0.001	0.002
Hen-day production, (%)	82.52	84.71	88.23	85.91	0.81	0.006	0.024
Feed intake, (g/hen/day)	106.8	104.6	106.7	106.1	1.68	0.997	0.664
Feed conversion ratio	2.106	1.932	1.851	1.920	0.033	0.002	0.006
Ammonia, g/100 g excreta	6.044	3.919	2.743	3.585	0.266	<0.001	<0.001

^1^ SEM: Standard error of the means.

**Table 4:** The effect of *Moringa oleifera* leaves on egg quality of laying hens during the late laying period.

Items	*Moringa oleifera* Leaves (g/kg)				SEM ^1^	*p* Value	
	0	3	6	9		Linear	Quadratic
68 weeks of age							
Haugh units, score	81.23	85.01	89.01	84.56	1.079	0.004	0.001
Shell thickness (mm)	0.378	0.396	0.408	0.399	0.043	0.006	0.014
Shell (%)	10.75	11.62	12.84	11.64	0.382	0.028	0.061
Albumen (%)	62.57	61.33	60.08	60.78	0.653	0.127	0.063
Yolk (%)	26.68	27.25	27.08	27.38	0.451	0.156	0.146
72 weeks of age							
Haugh units, score	82.13	86.11	89.51	85.76	1.081	0.001	0.001
Shell thickness (mm)	0.381	0.399	0.409	0.401	0.054	0.001	0.024
Shell (%)	10.85	11.73	12.88	11.74	0.883	0.013	0.032
Albumen (%)	62.67	61.21	60.04	60.56	0.991	0.063	0.451
Yolk (%)	26.48	27.16	27.18	27.7	0.654	0.076	0.236

^1^ SEM: Standard error of the means.

**Table 5:** The effect of *Moringa oleifera* leaves on serum metabolic profile of laying hens during the late laying period.

Items	*Moringa oleifera* Leaves (g/kg)				SEM ^1^	*p* Value	
	0	3	6	9		Linear	Quadratic
Cholesterol (mg/dL)	154.41	144.51	137.36	142.70	1.260	0.001	0.001
Triglycerides (mg/dL)	138.29	128.13	124.17	128.91	0.556	0.001	0.004
ALT (U/L)	29.69	25.50	21.51	22.97	0.824	0.001	0.001
AST (U/L)	67.79	61.56	57.01	61.19	0.992	0.001	0.001
Creatinine (mg/dL)	0.624	0.456	0.401	0.415	0.009	0.001	0.001
Uric acid (mg/dL)	4.253	3.574	2.710	3.490	0.207	0.001	0.001

^1^ SEM: Standard error of the means.

## Data Availability

Data presented in this study are available on fair request from the respective author.
